# 613. Violent Burn Injuries Are Associated with Sociodemographic Disparities and Increased ICU Requirements

**DOI:** 10.1093/jbcr/irag033.427

**Published:** 2026-04-06

**Authors:** Artur Manasyan, Sarah Wang, Karel-Bart Celie, Sarah A Stoycos, T Justin Gillenwater, Haig A Yenikomshian, Maxwell B Johnson

**Affiliations:** Los Angeles General Medical Center Regional Burn Center, Los Angeles, CA; Keck School of Medicine, University of Southern California, Los Angeles, CA; Division of Plastic and Reconstructive Surgery, Keck School of Medicine, University of Southern California, Los Angeles, CA; Keck School of Medicine, University of Southern California, Los Angeles, CA; Division of Plastic and Reconstructive Surgery, Keck School of Medicine, University of Southern California, Los Angeles, CA; Division of Plastic and Reconstructive Surgery, Keck School of Medicine, University of Southern California, Los Angeles, CA; Division of Plastic and Reconstructive Surgery, Keck School of Medicine, University of Southern California, Los Angeles, CA

## Abstract

**Introduction:**

Violent or intentional burn injuries represent a severe form of trauma often stemming from interpersonal conflicts and high-risk social circumstances. There is a paucity of literature examining the socioeconomic factors associated with violent burn injuries and how their clinical outcomes differ from non-violent burns. The objective of this study is to identify the sociodemographic factors associated with such injuries and evaluate their association with morbidity and mortality.

**Methods:**

A retrospective review was conducted for all patients admitted to a large urban burn center between 2012 and 2025. Admission records were screened to identify burns of violent origin. Patients identified as having self-inflicted burn injuries were excluded. Independent t-tests were used for continuous variables and chi-square tests for categorical variables, and regression analyses were performed to assess the relationship between violent burn etiology and clinical outcomes.

**Results:**

Of the total 4939 included patients, 213 (4.3%) had intentional burns. Patients in the violent burn cohort were younger (35.4 ± 20.1 vs. 38.9 ± 22.8 years, *p*=.03), more often Black (31.5% vs. 14.3%, *p*<.01), homeless (40.4% vs. 13.7%, *p*<.01), single/unmarried (73.2% vs. 55.6%, *p*<.01), and insured by Medicaid (75.1% vs. 54.2%, *p*<.01). Drug use was also more common among intentional burn patients (54.5% vs. 31.2%, *p*<.01). The violent burn cohort had more severe injury. Rates of inhalation injury (13.6% vs. 8.7%, *p*=.013) and larger affected total body surface area (TBSA) (8.0 ± 7.7 vs. 5.5 ± 15.3, *p*<.01) were present in this cohort. Multivariate regression analysis showed that violent burns, after controlling for age, sex, TBSA, and inhalation injury, were significantly associated with longer intensive care unit stay (coeff: 3.18, *p*=.031), more days requiring mechanical ventilation (coeff: 3.45, *p*=.026), and longer inpatient length of stay (coeff: 2.60, *p*=.02). Mortality was not significant (coeff: -0.01, *p*=.324).

**Conclusions:**

Patients with violent burns represent a vulnerable subgroup with higher social risk factors, greater injury severity, increased critical care needs, and longer inpatient stays. Providing comprehensive social services, including access to social workers, psychologists, and substance use treatment, may help target underlying socioeconomic and psychosocial vulnerabilities and reduce the risk of violent burn injuries.

**Applicability of Research to Practice:**

Patients with violent burn should be prioritized for social work, mental health, and substance use support to address underlying risks and improve recovery.

**Funding for the study:**

N/A.

